# New Insights for Native Production of MSP1_19_, the Disulfide-Rich C-Terminal Fragment from *Plasmodium falciparum* Merozoite Surface Protein 1

**DOI:** 10.1371/journal.pone.0057086

**Published:** 2013-02-22

**Authors:** Anne-Gaëlle Planson, J. Iñaki Guijarro, Alain F. Chaffotte

**Affiliations:** 1 Unité de RMN des Biomolécules, Institut Pasteur, Paris, France; 2 CNRS UMR 3528, Paris, France; Instituto de Ciências Biomédicas/Universidade de São Paulo - USP, Brazil

## Abstract

Malaria represents a major public health problem and an important cause of mortality and morbidity. The malaria parasites are becoming resistant to drugs used to treat the disease and still no efficient vaccine has been developed. One promising vaccine candidate is the merozoite surface protein 1 (MSP1), which has been extensively investigated as a vaccine target. The surface protein MSP1 plays an essential role in the erythrocyte invasion process and is an accessible target for the immune system. Antibodies to the carboxy-terminal region of the protein, named MSP1_19_, can inhibit erythrocyte invasion and parasite growth. In order to develop an effective MSP1_19_- based vaccine against malaria, production of an antigen that is recognized by protective antibodies is mandatory. To this aim, we propose a method to produce the disulfide-rich MSP1_19_ in its native conformation based on its *in vitro* oxidative refolding. The native conformation of the renatured MSP1_19_ is carefully established by immunochemical reactivity experiments, circular dichroism and NMR. MSP1_19_ can successfully be refolded *in vitro* as an isolated protein or as a fusion with the maltose binding protein. The possibility to properly fold MSP1_19_ *in vitro* paves the way to new approaches for high titer production of native MSP1_19_ using *Escherichia coli* as a host.

## Introduction

*Plasmodium falciparum* is the major cause of human malaria, an endemic disease that can quickly become life threatening if not treated. The World Health Organization estimates that malaria causes 300 to 500 million infections and over 1 million deaths each year almost exclusively among young children and pregnant women [1]. Although antimalarial treatments such as artemisinin combination therapies are widely used against *Plasmodium falciparum* infections, the parasites have developed resistance to a number of malaria drugs and there is thus a need to develop an effective vaccine. The RTS,S vaccine, which targets the circumsporozoite surface protein (pre-erythrocytic stage) is currently in phase 3 trials and has shown protection against *P. falciparum* malaria in *ca.* 50% of children and infants [2]. There is still, however, a major interest to develop a vaccine that targets the malaria blood stage. The blood stage malaria vaccine candidates are based on antigens that coat the surface of the merozoite, which is the red blood cells invasive form of the parasite. Immunization with such antigens should generate protective antibodies able to block invasion. The merozoite surface protein 1 (MSP1) is the most abundant protein on the surface of *P. falciparum* merozoites [3] and is one of the best characterized of many proteins on the merozoite surface that are being targeted for malaria vaccine development [4], [5]. MSP1 is essential during the invasion blood stage. The protein is synthesized in schizonts as a ∼190 kDa glycosylphosphatidylinositol (GPI) anchored protein that is processed by *P. falciparum* subtilisin 1 at the end of the schizogony into four polypeptides named p83, p42, p38 and p30. These fragments remain associated together on the parasite’s surface [6]. The C-terminal GPI moiety (p42) undergoes a secondary processing during the final stage of erythrocyte invasion by *P. falciparum* subtilisin 2, generating MSP1_33_ and MSP1_19_ [7]. The C-terminal fragment MSP1_19_, here named F19, remains attached on the parasite’s surface through its GPI anchor until the end of the intracellular cycle [8]. The F19 fragment is the target of protective antibodies that can block the parasite invasion of erythrocytes and the presence of anti-F19 antibodies in human sera correlates with the immunity against *Plasmodium* [9], [10]. The F19 fragment is one of the most promising antigens for a malaria vaccine and it has been used in the attempt for developing multi-antigen vaccines that are based on the fusion of multiple epitopes [11].

The native structure of F19 consists of two epidermal growth factor-like (EGF-like) domains, each containing three disulfide bonds [12]. The correct arrangement of disulfides that dictates the native fold is critical in an immunogenic context: most of the F19 B-cell epitopes appear to be non-linear and disulfide bond dependent as irreversible reduction of the disulfides abolishes protective-antibody recognition [13]. Moreover, the choice of the expression system, which may lead to F19 in different conformations, is of outmost importance for vaccine design: F19 has been produced in the *Escherichia coli* cytoplasm, yeast and baculovirus-infected-cell systems, but recombinant proteins expressed in *E. coli* or in yeast did not confer any protective efficacy in primates or the latter was highly inconsistent compared with the recombinant F19 produced in the baculovirus expression system [14]. Also, in blind tests of immunogenicity and of functional activity (protection) of the antibodies obtained after rabbit immunization, F19 produced in the baculovirus system performed significantly better than F19 produced in the *E. coli* cytoplasm [11].

Nevertheless, the baculovirus system is onerous and cost efficient production is a major issue to consider for a malaria vaccine. Because of its low cost and possible high yields, whenever the protein can be obtained, *E. coli* remains the choice of excellence for recombinant protein production. Because the correct disulfide bond formation of F19 is required for its immunogenicity [15], the *E. coli* cytoplasm, which has a reducing potential that hampers cysteine oxidation, is not suitable for producing disulfide-containing proteins. As previous attempts of F19 *in vitro* oxidative folding under many different conditions had failed to produce it in its native conformation, F19 bacterial production was carried out in the *E. coli* periplasm [16], which provides an oxidative environment and a machinery of disulfide isomerases. F19 was successfully produced in its native form in the periplasm of *E. coli* when fused to the maltose binding protein (MBP) but obtained in a non-native heterogeneous soluble form in the absence of MBP. This work revealed the essential role played by MBP in the F19 oxidative folding *in vivo* and enabled to consider a new alternative for producing the F19 vaccine candidate properly folded. However, periplasmic expression led to low protein yields. With the aim of exploring novel approaches for production of native F19 from *E. coli*, in this work we studied the *in vitro* oxidative refolding of F19 fused to MBP or as an isolated protein fragment. Structural and immunoreactive properties of the resulting F19 were analyzed and compared with those of the F19 produced in insect cells used as a reference for the native conformation. Here, we propose a novel method to efficiently fold F19 into its native conformation *in vitro*, paving the way to use *E. coli* as a production host.

## Materials and Methods

### Strains and Plasmids

*E. coli* strains PM9 (*recA1 supE44 endA1, hsd R17, gyr A96, ThiΔ(lac-proAB),ΔmalE444)* and NS2 [17], as well as the plasmids encoding for the protein MBP-F19 fusion protein were previously described [16].

### Protein Nomenclature and Description

MBP-F19 was produced in the *E. coli* periplasm and refers to the protein fusion containing a factor Xa cleavage site between MBP and F19 (Table 1). The F19 moiety extracted from this fusion is named F19ec (residues 1597–1726 of MSP1 [18]); it contains a 25-residue-long N-terminal extension (including nine residues from the expression vector) and a C-terminal lysine compared to the sequence of F19 obtained from baculovirus-infected insect cells (F19bac, residues 1613–1726 of MSP1 as determined by N-terminal sequencing and mass spectrometry). Although the same sequence was cloned for *E. coli* and insect cells expression, the protein obtained from insect cells is cleaved *in vivo* and is consequently shorter. Residues 1597–1612 of MSP1 were included in the F19ec construct because this sequence contains the secondary proteolytic processing site of MSP1 (MSP1_42_ cleavage into MSP1_33_ and F19) and antibodies with epitopes in this specific region may block parasite invasion of red blood cells [19]. F19ec will be named F19ec,rf when it has been refolded *in vitro* within the fusion protein MBP-F19 and subsequently isolated by proteolytic cleavage, or F19ec,ri when it has been renatured as an isolated protein (i.e. not fused to MBP). F19bac (residues 1613–1726 of MSP1 and a C-terminal hexa-histidine tag for purification purposes), which was used to determine the X-Ray structure of F19 and has been extensively studied in immunization and immunoreactivity studies, is used here as a reference for the native conformation of F19 [12].

**Table 1:** Protein nomenclature.^a^

Name	Description
MBP-F19	Fusion protein MBP-F19 produced in *E. coli* periplasm with a native F19 moiety [16]; contains a factor Xa cleavage site between MBP and F19.
F19ec	Native F19 isolated from MBP-F19 by proteolytic cleavage with factor Xa.
F19ec,rf	F19 renatured fused to MBP and then isolated from the fusion.
F19ec,ri	F19ec, which was denatured and renatured *in vitro* in its isolated form (*i.e.* not in fusion with MBP).
F19bac	F19 fragment produced in baculovirus-infected insect cells
F19bac,r	F19bac denatured and renatured *in vitro*.

a The sequence of the F19 fragments is derived from PfIT_09_02 (1185913–1186197) of the PlasmoDB database (http://plasmodb.org).

### Protein Expression and Purification

MBP-F19 periplasmic expression was induced by IPTG (1 mM) in *E. coli* strain NS2 grown at 30°C in 2YT [20] medium supplemented with ampicillin (100 µg/ml). After centrifugation, the cell pellet was resuspended in buffer A (20 mM Tris, 100 mM NaCl, pH 8.0) with 1 mg/ml polymyxine B sulfate to obtain the periplasmic fraction. MBP-F19 protein was purified using an amylose agarose column (New England Biolabs Inc.). The F19 moiety was isolated from the fusion using factor Xa (New England Biolabs Inc.) and purified by two successive chromatographies: first MBP and residual MBP-F19 were removed with an amylose agarose column and second, factor Xa was removed by means of an immunoadsorbent column specific for F19 (antibody G17.12, gift from F. Nato, Institut Pasteur).

### Protein Concentration

The protein concentrations were determined by UV spectrophotometry using the following molar extinction coefficient at 280 nm: 70080 M^−1^.cm^−1^ for oxidized MBP-F19, 69330 M^−1^.cm^−1^ for reduced MBP-F19, 3730 M^−1^.cm^−1^ for oxidized F19 and 2980 M^−1^.cm^−1^ for reduced F19 [21]. The extinction coefficients used in the presence of 3 M guanidinium hydrochloride (GdnHCl) were 68000 M^−1^.cm^−1^ for MBP-F19 and 3280 M^−1^.cm^−1^ for oxidized F19.

### Protein Denaturation

MBP-F19, F19ec and F19bac were unfolded by incubation at 4°C for at least 12 hours in 6 M GdnHCl prepared in PBS at pH 7.5 supplemented with 0.1 M 2-mercaptoethanol, and then dialyzed against 3 M GdnHCl in PBS at pH 5.8 at 4°C to maintain the proteins in their denatured and reduced state.

### Iodoacetamide Labeling

The redox state of reduced and denatured F19 was assessed using iodoacetamide labeling. Denatured F19 (100 µg/ml in 6 M GdnHCl, 0.1 M 2-mercaptoethanol, pH 7.5) was diluted (final concentration 17 µg/ml) into 0.2 M iodoacetamide/6 M GdnHCl (Sigma-Aldrich, France). The alkylation reaction was carried out for 1 min at room temperature in the dark. Cysteine alkylation was analyzed by using ProteinChip arrays technology (Bio-rad, USA) coupled with mass spectrometry (SELDI-TOF-MS, Microsequencing and Mass Spectrometry Facility, Institut Pasteur). Mass spectrometry indicated a difference of 695 Da between fully oxidized F19ec and iodoacetamide-labeled denatured and reduced F19ec, corresponding to 12 labeled cysteines (58 Da per acetamide group). Furthermore, in a control experiment on non-reduced F19ec no mass difference was observed after iodoacetamide reaction indicating that the labeling occurred selectively on cysteine groups. Therefore the twelve cysteines of F19 were reduced under the denaturing conditions used.

### Protein Renaturation

Protein renaturation was carried out at 25°C by stepwise additions under continuous magnetic stirring of denatured and reduced protein into the renaturation buffer, increasing progressively the concentration of protein to avoid high local concentrations of unfolded and folding protein that could favor aggregation. Ten aliquots (0.2 ml) of denatured protein (280 µM in GdnHCl 3 M pH 5.8) were added to PBS pH 7.5 (40 mL) supplemented with the redox couple 0.5 mM GSSG/1.0 mM GSH every 90 min under shaking. Final concentrations were 14 µM for the protein and 0.15 M for the denaturant. The F19 moiety (hereafter named F19ec,rf) was isolated from the fusion protein by proteolytic cleavage with factor Xa and purified as described above.

Denatured F19ec and F19bac were renatured using the same protocol that was used for F19ec,rf. Initial (denatured, 3 M GdnHCl) and final (renatured, 0.08 M GdnHCl) concentrations of F19ec were 100 µM and 3 µM, respectively. For F19bac the initial and final concentrations were 280 µM (3 M GdnHCl) and 19 µM (0.19 M GdnHCl), respectively. The F19 fragments renatured *in vitro* in the absence of MBP, are called F19ec,ri and F19bac,r. Following the refolding step, F19ec,ri and F19bac,r were filtrated through a PD10 column (GE Healthcare Lifesciences, USA) to remove oxidized and reduced glutathione as well as residual GdnHCl, dialyzed against 50 mM ammonium bicarbonate and lyophilized.

### Immunoreactivity

The immunoreactivity of F19 was analyzed by competition ELISA [22]. Affinity constants of F19 versus three monoclonal antibodies G17.12, D11.4 and D12.8 (gift of F. Nato, Institut Pasteur) were determined in solution as previously described in Planson *et al.* [16].

### Circular Dichroism

Far-UV circular dichroism (CD) spectra were acquired on an Aviv215 spectropolarimeter between 185 nm and 260 nm with a 1.5 nm bandwidth and 0.5 nm steps. F19 samples (0.5 mg/ml) were prepared in 50 mM ammonium bicarbonate pH 7.5 by dialysis. Baselines were recorded using the dialysis buffer and subtracted from the sample spectrum. Quantitative secondary structure analysis from normalized spectra was done using CDPro [23].

### NMR

Lyophilized proteins were dissolved in 20 mM deuterated sodium acetate, 10% D_2_O, pH 4.0 and centrifuged at 17600 g for 30 min at 4°C to remove any possible aggregates. The concentrations of F19ec,rf, F19ec,ri and F19bac,r were 0.4 mM, 0.2 mM and 0.7 mM, respectively. The experiments were conducted on an Inova 500 or an Inova 600 spectrometer equipped with a cryoprobe (Varian Inc. Palo Alto, CA). Data were processed and analyzed using the softwares Vnmr 6.1C (Varian Inc.) and NMRView 5.0.3 [24]. Nuclear Overhauser effect spectroscopy (NOESY) [25] and total correlation spectroscopy (TOCSY) [26], [27] spectra were acquired at 35°C with 2048 data points in the direct dimension, 256 t_1_ increments, and 16 or 64 transients per t_1_ increment. Spectra were recorded using a spectral window of 12 ppm and a recycling delay of 1.6 or 2.2 s. Solvent suppression was achieved by means of the watergate pulse scheme [28], [29]. Mixing times in NOESY and TOCSY experiments were 120 and 70 ms, respectively.

## Results

### Obtention of F19 Fragments

**Figure 1:**
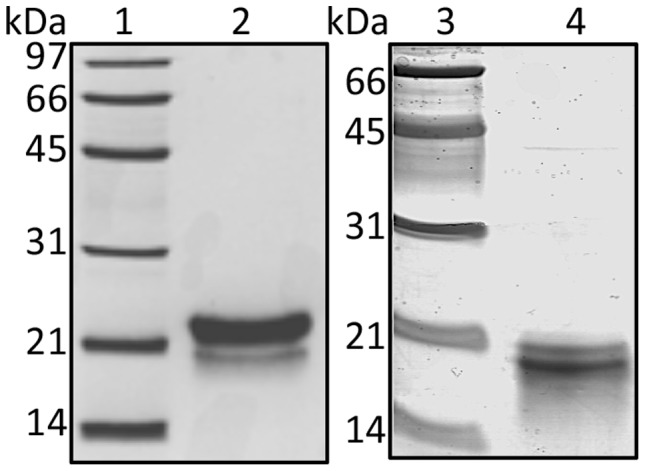
SDS-PAGE of purified F19 fragments. Samples were denatured in 2% SDS in the presence of 5% 2-mercaptoethanol before being subjected to 12% SDS-PAGE. Lanes 1 and 3: molecular weight markers; lane 2: F19bac; lane 4: F19ec.

Seventeen mg of pure MBP-F19 containing ca. 4 mg of F19ec were obtained per liter of bacterial culture. After cleavage by factor Xa and further purification, *ca.* 3 mg (per liter of culture) of pure F19ec were obtained. F19bac produced in insect cells [14] was a generous gift from Shirley Longacre (Institut Pasteur). Figure 1 shows the SDS-PAGE patterns of purified F19ec and F19bac. It should be noted that F19ec, which comprises a 25 aminoacid extension at its N-terminal end migrated faster than F19bac. This difference in mobility is likely due to a global higher proportion of hydrophobic residues in F19ec resulting in a higher ratio of SDS bound per peptide chain.

### *In vitro* Folding of the MBP-F19 Fusion Protein

The oxidative renaturation using the GSH/GSSG redox couple is a slow process. Before refolding completion, the initially unfolded protein goes through intermediate states prone to aggregation, the extent of which is highly concentration dependent. Thus, in order to optimize the refolding yield, we used a pulsed renaturation procedure (see Materials and Methods) aimed at keeping low the concentration of aggregation-prone species while enriching the solution in refolded protein by stepwise additions.

The final conformational state of the F19 moiety, which lacks specific structural probes such as tryptophan fluorescence, was evaluated by testing its immunoreactivity against monoclonal antibodies that preferentially recognize epitopes in the native conformation [17]. To this end, we determined the affinity constant in solution of three different antibodies (G17.12, D11.4, D12.8) for F19 folded *in vitro* within the MBP fusion and against fully reduced MBP-F19, and compared them with the values previously obtained for the native protein produced in the *E. coli* periplasm [17]. The affinity constants of native F19 were 2.5 to 3.8 higher than those of the denatured protein depending on the antibody (Table 2). These differences are significantly greater than the experimental error. Furthermore, the affinity constants of the three antibodies against the refolded and native proteins are very similar hence the refolded protein is better recognized by the three antibodies than the unfolded and reduced protein. This result indicates that (i), the refolded protein contains native epitopes and may thus be properly folded and (ii) the refolding is efficient. Indeed, if the refolded sample contained a mixture of unfolded and native protein, the affinity constants of the refolded F19 samples should be comprised between those characteristic of the unfolded and native conformations. In addition, no aggregates were observed during the folding step. In summary, immunoreactivity experiments suggest that the F19 moiety is natively folded and that the folding is efficient.

**Table 2:** Solution equilibrium association constants of F19-antibody interactions.^a^

	nativeMBP-F19^b^	denaturedMBP-F19^c^	renaturedMBP-F19^d^	R_N/D_ e
G17-12	7.8×10^9^	2.3×10^9^	6.6×10^9^	3.4
D11-4	1.3×10^8^	0.3×10^8^	1.4×10^8^	3.8
D12-8	5.7×10^8^	2.4×10^8^	10.6×10^8^	2.4

a Association constants (K_A_) were determined at 25°C in PBS-Tween buffer pH 7.4 supplemented with 0.02% bovine serum albumin. K_A_ values are expressed in M^−1^. The experimental error represents 20–25% of the corresponding K_A_ value.

b MBP-F19 fusion protein natively produced in *E. coli* periplasm [16],

c denatured and reduced fusion protein,

d fusion protein renatured *in vitro*,

e ratio of the K_A_ values obtained for the native and denatured fusion protein.

### Structural Characterization of F19ec,rf after Renaturation of MBP-F19

After MBP-F19 renaturation as described above, the F19 moiety (F19ec,rf) was obtained by cleavage of the fusion protein with factor Xa, purified and its structure was characterized in detail by NMR. Indeed, NMR spectra can be used as fingerprints of a given structure. The pattern of peaks in NOESY spectra, which arise from dipolar interactions between protons close to each other (≤5 Å apart), is particularly sensitive to even slight changes in structure. TOCSY (through bond correlation) and NOESY (through space correlation) NMR experiments were recorded, and NMR spectra of two references for the native conformation of F19 were used. The references were F19bac obtained from baculovirus infected cells, of which the structure was solved by X-ray crystallography [12], and F19ec obtained in its native conformation from the periplasmic expression of MBP-F19 in *E. coli* [16] as evidenced by the superimposition of its NMR spectra with that of F19bac.

**Figure 2:**
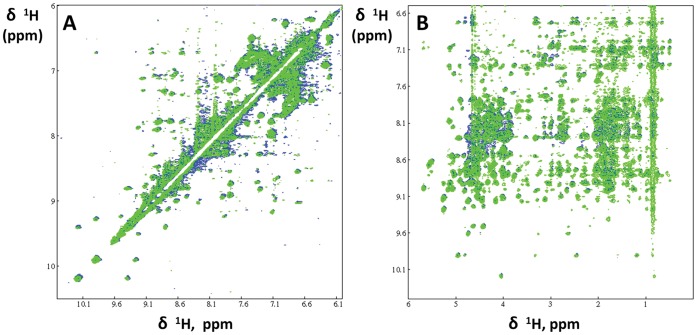
NOESY spectra of F19ec and F19ec, rf. Two regions of NOESY spectra of F19ec (blue) and F19ec,rf isolated from renatured MBP-F19 (in green). **A** Low field region and **B** amide/aromatic *versus* aliphatic regions.

TOCSY (not shown) and NOESY ^1^H NMR spectra of the fragment were recorded and carefully compared with the equivalent spectra obtained for native F19ec under the same experimental conditions (Figure 2). F19ec,rf spectra superimpose very well with the equivalent spectra of native F19ec, unambiguously indicating that the *in vitro* pulsed renaturation method produced native F19. In addition, no sign of unfolded protein was observed in the spectra. Given that NMR can detect minor species with a relative population of at least 5%, we can conclude that native F19ec,rf represented at least 95% of the sample. The yield of folded F19/initial denatured F19 after the refolding and purification steps was at least 70%, which is a relatively good yield for an *in vitro* oxidative refolding of a disulfide rich protein.

### *In vitro* Folding of Isolated F19 (F19ec,ri)

Although *in vitro* folding of MBP-F19 lead to the native conformation of the F19 moiety, we also investigated the folding of isolated F19 since proteolytic cleavage and the subsequent purification steps needed to get rid of MBP and the protease might represent an economical limitation in vaccine development. Noteworthy, previous attempts to fold isolated F19 *in vitro* and *in vivo* in *E. coli* were unsuccessful [16]. Isolated F19 was denatured and reduced and renatured following the same protocols used for the MBP-F19 fusion. Both proteins called F19ec,rf (obtained from MBP-F19 renaturation) and F19ec,ri (obtained from isolated F19 renaturation) are strictly identical in their sequence but differ in the way they were obtained (Table 1).

**Figure 3:**
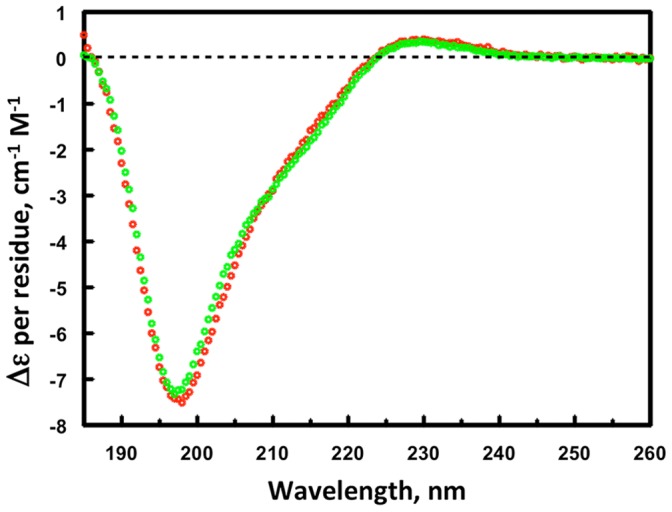
Circular dichroism spectra of F19ec,ri and F19bac. Superimposition of the circular dichroism spectra of *in vitro* renatured F19ec,ri (red) and of F19bac (green) used as a reference for F19 native conformation.

**Figure 4:**
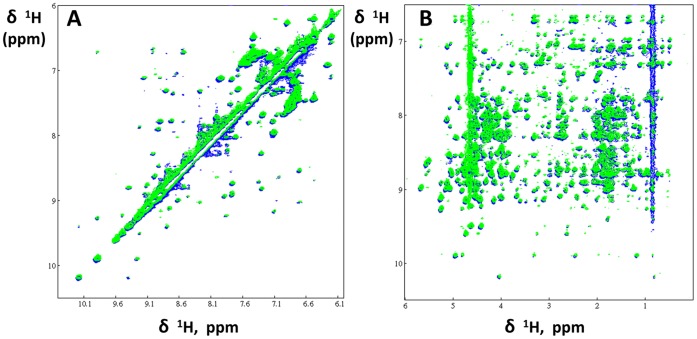
NOESY spectra of F19ec,rf and F19ec, ri. Two regions of NOESY spectra of F19ec,rf, which was isolated from renatured MBP-F19 (blue) and F19ec,ri (green), which was denatured and refolded *in vitro* in its isolated form. **A** Low field region and **B** amide/aromatic *versus* aliphatic regions.

The F19ec,ri conformational state was analyzed by circular dichroism and NMR. The CD spectrum of F19ec,ri superimposes perfectly with that of F19bac used as a reference (Figure 3), indicating that F19ec,ri shows a native secondary structure content and that the N-terminal extension in F19ec,ri does not contribute significantly to its far-UV spectrum. Furthermore, TOCSY and NOESY spectra of F19ec,ri are very similar to the spectra of F19ec and F19ec,rf (Figure 4). Therefore, F19ec,ri displays the same structure as F19ec and is natively folded. Interestingly, F19 in its isolated form can acquire its native conformation *in vitro* but not *in vivo* in *E. coli*, indicating that the assistance of MBP established for *in vivo* folding of F19 is not essential for its oxidative folding *in vitro*.

### *In vitro* Folding of F19bac

**Figure 5:**
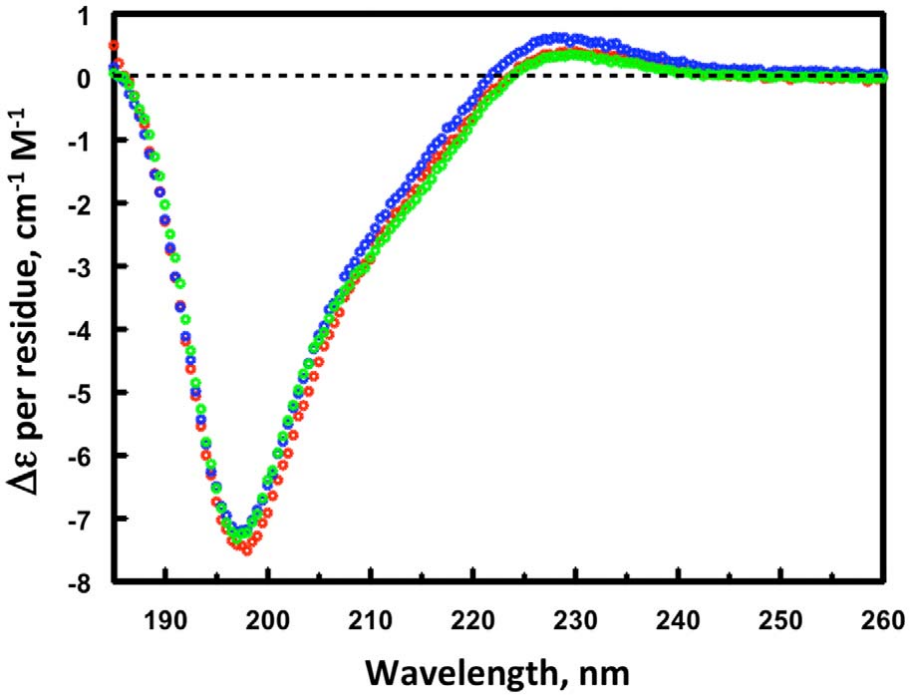
Circular dichroism of F19ec,ri, F19bac,r and F19bac. Superimposition of the circular dichroism far-UV spectra of F19ec,ri (red), F19bac,r (blue) and F19bac (green).

**Figure 6:**
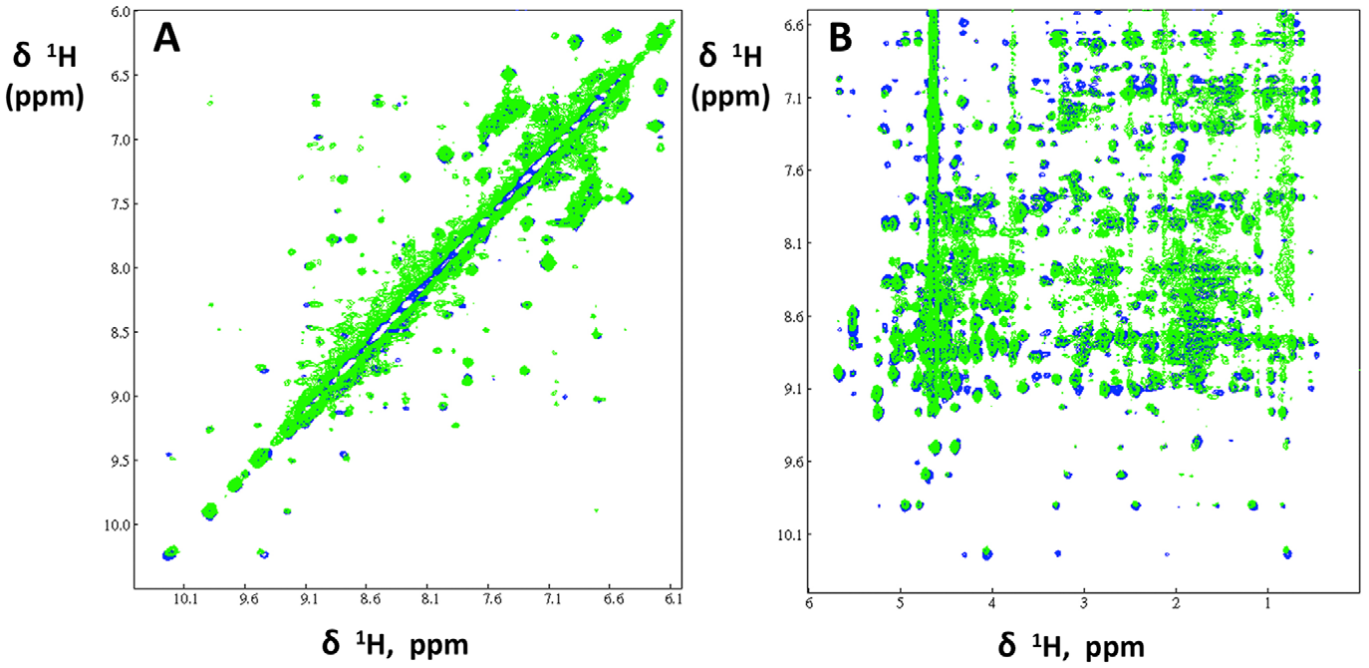
NMR NOESY spectra of F19bac et F19bac,r. Superimposition of two areas of the NOESY spectra of F19bac (blue), which serves as a reference for the native state, and of F19bac,r (green), which was denatured and renatured *in vitro*. **A** Low field region and **B** amide/aromatic *versus* aliphatic regions.

**Figure 7:**
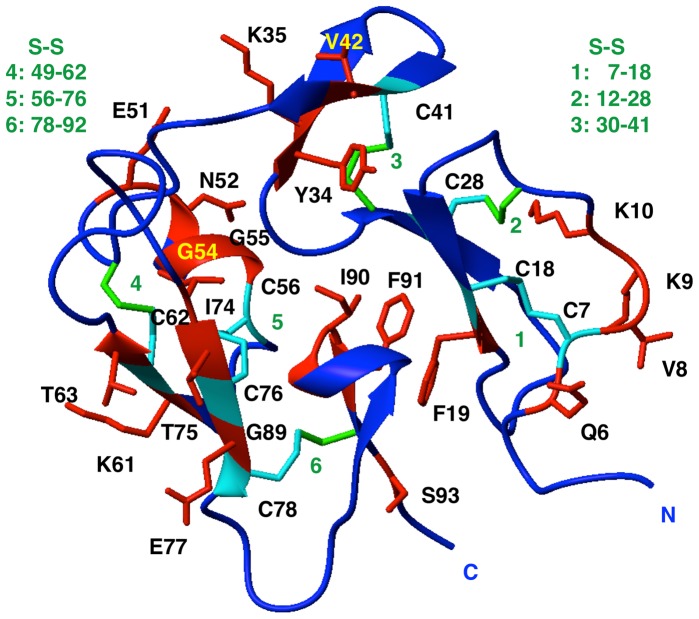
F19 structure highlighting residues identified by NMR. Ribbon representation of the F19 structure (F19bac) solved by X-ray crystallography (PDB code 1OB1). Assigned residues that show a dipolar interaction in the NOESY spectra of F19 in agreement with a short distance in the structure are highlighted in red or in cyan in the case of assigned cysteines.

As stated previously, F19ec has an N-terminal extension (25 residues) in comparison with F19bac. Although previous NMR studies had shown that the N-terminal residues of the extension in F19ec are not structured [16], it was important to assess whether this extension plays a role on the efficiency of the F19 *in vitro* renaturation. We thus denatured and reduced F19bac and renatured it using the same oxidative folding protocol used for F19ec,ri and examined its conformation by far-UV CD and NMR. The CD spectrum obtained for renatured F19bac (F19bac,r) was effectively identical to the spectrum of native F19bac (Figure 5) and spectra deconvolution using CDPro [23] indicated a very similar secondary structure content (Table 3). Furthermore, renatured F19bac and native F19bac showed effectively the same NOESY (Figure 6) and TOCSY (not shown) spectra. Together, these results indicated that F19bac can also be correctly refolded *in vitro* and that the N-terminal extension of F19ec does not participate in the folding of F19 *in vitro*. Furthermore, on the basis of the published chemical shifts of the F19 fragment obtained from yeast and used for structure determination by NMR [30], by focusing on well resolved HN resonances and using through-bond as well as intra- and inter-residue through-space proton-proton connectivities, we unambiguously assigned signals in F19ec and F19bac TOCSY and NOESY spectra for the following 30 residues: Q6, C7, V8, K9, K10, C18, F19, C28, Y34, K35, C41, V42, E51, N52, N53, G54, G55, C56, K61, C62, T63, I74, T75, C76, E77, C78, G89, I90, F91, S93 in F19bac numbering (Figure 7). These residues are spread throughout the entire sequence and structure of F19. Importantly, the signals of seven cysteine residues (C7, C18, C28, C41, C62, C76 and C78) involved in the four disulfide bridges, of residues contiguous to the latter cysteines (Q6, V8, F19, V42, K61, T63, T75 and E77), and of residues I90 and F91, located in the hydrophobic core at the interface of the EGF modules, were assigned and showed nOes (short distances) in agreement with the native structure of F19bac. The signals identified are identical in the spectra of F19ec,rf, F19ec,ri, F19bac,r and F19bac. All together these results unambiguously demonstrate the native conformation of the *in vitro* refolded protein fragments.

**Table 3:** Secondary structure content of F19.^a^

	α−helix	β−sheet	Turn	Random coil
F19ec,ri	5%	29%	23%	43%
F19bac,r	4%	30%	23%	43%
F19bac	4%	31%	22%	43%
F19bac (X-Ray)	7%	30%	18%	45%

a The results from quantitative analysis of circular dichroism spectra of F19ec,ri – F19bac,r and F19bac are shown in percentage of secondary structures. Analyses were performed using the CONTINLL method from the CDPro software [23]. The secondary structure content of the X-Ray structure of F19 is also indicated [12].

## Discussion

To summarize, we showed that the MSP1 F19 fragment, a disulfide rich malaria vaccine candidate, can be obtained in its native conformation by *in vitro* oxidative refolding fused to MBP or as an isolated fragment. The method of gradual addition of unfolded protein to the folding buffer (pulsed renaturation) was essential to obtain the F19 fragment in its native conformation: previous attempts to properly fold the F19 fragment *in vitro* under a large array of experimental conditions (different concentrations of reduced and oxidized glutathione, the presence or absence of folding adducts such as urea or nondetergent sulfobetaines, various pHs and temperatures) had failed to produce native F19 [16].

The capacity to correctly fold F19 *in vitro* with high yields represents an important step forward in native F19 production from *E. coli* because cytoplasmic expression instead of periplasmic expression, which resulted in low yields of native MBP-F19, can be considered. F19 can be expressed in the cytoplasm in high amounts fused to MBP or as an isolated protein and recuperated from the soluble and/or insoluble fraction, denatured and refolded *in vitro*. For large-scale production, fusing MBP to F19 could represent an advantage for purification. Using isolated F19 would avoid the proteolytic and downstream purification steps.

Although it is now commonly thought that subunit vaccines against blood stages of malaria will not suffice to eliminate infection, a highly efficient multivalent malaria vaccine will must likely incorporate blood stage antigens [31], [32]. F19 is an important antigenic region of MSP1, which has long been considered a promising blood stage *P. falciparum* vaccine candidate. The F19 fragment presents the advantage that it shows a low level of polymorphism (only 5 dimorphic sites located on the surface of the fragment) in an otherwise highly variable protein, allowing to envision a complete coverage of polymorphisms with only two different preparations of the antigen [33]. Importantly, significant correlations between levels of antibodies against F19 and protection from malaria have been reported and a recent analysis indicated a reduction in malaria risk for individuals with anti-F19 antibodies [34], [35]. Immunoreactivity studies with sera of malaria immune individuals and vaccination tests on primates have shown that the conformation of F19, and particularly the formation of the correct disulfides, is critical for the elicitation of protective antibodies *in vivo*. In this work, we have shown that native F19 can be obtained from i*n vitro* folding and thus have contributed to the development of novel routes for F19 malaria vaccine-candidate production.
